# Two-Eyed Seeing in LGBTQ+ aging research: critical lessons from research with Two-Spirit and Indigenous LGBTQ+ people aging with HIV

**DOI:** 10.3389/fpubh.2026.1965121

**Published:** 2026-10-21

**Authors:** Chase M. Bryer, Jordan P. Lewis, Alexandra B. Collins, Madina Agénor, Trudie Jackson, Itai Jeffries, Paul Goulet, Nathan Lachowsky, Julia McQuoid, Jeff Berry, Sherrill Wayland, Jeffrey Proulx

**Affiliations:** 1 Center on Aging, University of Hawai'i at Manoa, Honolulu, HI, United States; 2 Department of Epidemiology, Tufts University, Medford, MA, United States; 3 Department of Behavioral and Social Sciences, Brown University, Providence, RI, United States; 4 College of Doctoral Studies, Grand Canyon University, Phoenix, AZ, United States; 5 The Raven Collective, LLC, Grayslake, IL, United States; 6 Community Engaged Research Council, Providence-Boston Center for AIDS Research, Providence, RI, United States; 7 School of Health Sciences, University of Northern British Columbia, Prince George, BC, Canada; 8 TSET Health Promotion Research Center and Department of Family and Preventive Medicine, The University of Oklahoma Health Sciences, Oklahoma City, OK, United States; 9 The Reunion Project, Chicago, IL, United States; 10 Special Initiatives and Partnerships, SAGE, New York City, NY, United States

**Keywords:** HIV and aging, Indigenous LGBTQ+, indigenous research methods, Two-Eyed Seeing, Two-Spirit, decolonizing methodologies, responsive care

## Abstract

**Background:**

Dominant approaches to aging and public health research are often misaligned with Indigenous understandings of health and well-being, particularly in their limited attention to cultural, spiritual, and relational dimensions. Two-Eyed Seeing integrates the strengths of Indigenous and Western knowledge systems, offering a framework for addressing health disparities and advancing health equity. This approach has informed culturally responsive health strategies, including those for Indigenous people with HIV.

**Objectives:**

This article critically reflects on the application of Two-Eyed Seeing in a study involving Indigenous Two-Spirit and LGBTQ+ (2S/LGBTQ+) people aging with HIV. We offer methodological reflections, lessons learned, and insights into how this approach can enhance HIV and aging research, practice, and health equity.

**Methods:**

Drawing from participant insights, Community Advisory Board (CAB) feedback, community observations, and researcher reflexivity, we critically examined the application of Two-Eyed Seeing throughout the research process, including relationship building, research design, data collection, analysis, and interpretation.

**Findings:**

Through reflexive engagement, we generated three key methodological insights: (1) Two-Eyed Seeing challenged Western public health narratives that characterize Indigenous 2S/LGBTQ+ communities as “hard to reach,” emphasizing relational accountability and trust building; (2) engagement with Indigenous Knowledge Holders and CAB members fostered transformative mentorship, strengthening the researcher’s community connectedness and understanding of relational accountability; and (3) explicit and ongoing reflexivity helped interrogate and disrupt traditional power dynamics between the “researched” and the researcher, fostering more reciprocal and community-centered knowledge production.

**Conclusion:**

Two-Eyed Seeing offers a meaningful framework for advancing culturally responsive HIV and aging research with Indigenous 2S/LGBTQ+ communities. Beyond integrating knowledge systems, this approach emphasizes relational accountability, mentorship, and critical reflexivity as essential to challenging harmful research practices and addressing power imbalances. These insights may offer practical considerations for researchers seeking to advance health equity through culturally grounded, community-engaged approaches.

## 1 Introduction

Colonialism, systemic racism, and the ongoing occupation of Indigenous lands have deeply eroded trust among Indigenous peoples involved in Western aging and public health research, as these systems have historically exploited, harmed, and sought to invisibilize^1^ Indigenous communities (1–3). This underscores the need to conduct research “in a good way” by honoring tradition, spirit, and the guidance of Indigenous Elders to ensure ethical, culturally grounded, and community-driven approaches (4, 5). Two-Eyed Seeing offers a pathway for reconciliation by positioning Indigenous knowledge on equal footing with Western knowledge, creating space for ethical collaboration that resists assimilation and instead prioritizes Indigenous sovereignty, self-determination, and the restoration of epistemic authority (6). Two-Eyed Seeing refers to the concept of learning to see from one eye with the strengths of Indigenous knowledges and ways of knowing, and from the other eye with the strengths of Western knowledges and ways of knowing (7, 8). The term has roots in Mi’kmaq tradition, and is an adaptation of *Etuaptmumk*, which translates in English to “the gift of multiple perspectives” (9). The concept was brought to the health sciences by Mi’kmaq elders Albert and Murdena Marshall and Dr. Cheryl Bartlett (10). Since its inception, it has received growing attention within the field of Indigenous health research.

Two-Eyed Seeing has been used as a tool for introspection, challenging researchers, clinicians, and others engaging with Indigenous communities to critically examine how Indigenous ways of knowing have been silenced within Western medical contexts (11). Notably, Two-Eyed Seeing has been viewed as a tool to resist the positivist paradigms that have historically harmed Indigenous communities (12). Positivist research paradigms, rooted in “objective truth” have often dismissed or invalidated Indigenous ways of knowing and being while reinforcing colonial power dynamics that ignore the holistic views of wellness within Indigenous communities, which encompass spiritual, emotional, mental, and physical health factors, involving the past, present, and future generations (13). Through emphasizing respect, the coexistence of multiple worldviews, and the strength inherent within Indigenous communities, Two-Eyed Seeing holds the potential to disrupt harmful, damage-centered research practices and challenge mainstream Western research practices, which frequently “diminish personhood or sovereignty, or (re)humiliate when circulated” (14). Two-Eyed Seeing is especially advantageous as Indigenous ways of conducting research have long been silenced, dismissed, or forced to conform to Western knowledge systems, often leading to their dilution rather than true integration (15). Though mainstream approaches such as Community-Based Participatory Research (CBPR) have been successful in promoting culturally responsive research with Indigenous people (16, 17, 44), Two-Eyed Seeing is distinctive in that it emerged within a Mi’kmaq context, is thus rooted in Indigenous understandings of relationality and ways of knowing (10). It also transcends disciplinary and academic borders, and is not limited to mere ontology, epistemology, methodology, or axiology; rather, it operates fluidly and guides all aspects of life (18).

Despite its wide adoption within Indigenous HIV research in Canada, recent reviews have highlighted inconsistencies in how researchers interpret and apply Two-Eyed Seeing with Indigenous peoples, emphasizing the need for thorough documentation of its use in research to maximize its benefit to communities (18–20). Therefore, this article aims to contribute to this call within the field by detailing the application of Two-Eyed Seeing in a study exploring aging trajectories among Indigenous 2S/LGBTQ+ people aging with HIV across the United States (US) and Canada. Drawing from field notes and participant and Community Advisory Board (CAB) observation, this article offers critical reflections, lessons learned, and future recommendations for employing Two-Eyed Seeing in aging and public health research, with a focus on increasing its uptake in HIV research involving Indigenous people. Notably, this study represents the first known use of Two-Eyed Seeing in a US and transnational context, specifically in research involving Two-Spirit and Indigenous LGBTQ+ (2S/LGBTQ+) people with HIV.

### 1.1 How has Two-Eyed Seeing been used in Indigenous HIV research?

Two-Eyed Seeing has increasingly been utilized in HIV-related research, particularly among Indigenous people with HIV in Canada. Applications of this method have documented meanings of cultural safety in home and community care settings with Indigenous older adults with HIV (19), impacts of distance on HIV care provision (21), and holistic, community-driven strategies for sexual health and STI/HIV prevention in the Canadian Arctic through the use of storytelling (22). Additionally, Two-Eyed Seeing was applied in a national Canadian survey in 2021 (Tracks survey) involving over 2,383 participants, including 997 Indigenous people who inject drugs, further demonstrating its broad applicability in addressing diverse aspects of HIV prevention and care (23). The 2021 Tracks survey exemplifies the application of Two-Eyed Seeing in quantitative research by engaging Indigenous people with lived experience to identify key findings, develop community-driven themes, and create culturally relevant infographics on access to harm reduction and healthcare services, including HIV and hepatitis C care and HIV pre-exposure prophylaxis (PrEP) (23). Each of these examples has had a positive impact on Indigenous people with HIV, highlighting the effectiveness of Two-Eyed Seeing in addressing diverse aspects of HIV prevention and care.

## 2 Methods

### 2.1 Two-Eyed Seeing as a framework for conceptualizing qualitative Indigenous 2SLGBTQ + aging and HIV study

During study conceptualization, Two-Eyed Seeing proved instrumental, resonating with previous findings that suggest this framework reshapes the questions posed in Indigenous health research (12). It was important for the first author (*and for any researchers utilizing Two-Eyed Seeing*) to reflect deeply on the historical, cultural, and political forces that impact the Indigenous community to who they are accountable; in this case, Indigenous 2S/LGBTQ+ people with HIV. The first author also recognized how the prevailing notion of “successful aging” often marginalizes Indigenous 2S/LGBTQ+ people, as well as people aging with chronic conditions like HIV, by excluding diverse aging experiences and perpetuating colonial and ableist narratives about whose lives are deemed worthy and less worthy (24–30).

Guided by Two-Eyed Seeing, research questions for the original qualitative study were designed to elicit stories that counter the dominant Western models of “successful aging,” exploring culturally defined aging trajectories and what it means to *age well* with HIV. The choice to focus on aging “well” was deliberate and reflected the greater spirit of the Two-Eyed Seeing approach by honoring the strengths inherent within Indigenous communities that are often ignored within the realm of Western health sciences research.

### 2.2 Applying Two-Eyed seeing principles

The study applied Two-Eyed Seeing principles, drawing on Jeffery et al. (31) review, which identified key practices for successful implementation of Two-Eyed Seeing in medical research, such as declaring author positionality, communicating group interpretations, fostering relationship building, including Indigenous advisory committees and Knowledge Holders, utilizing traditional knowledge gathering techniques, and engaging in collaborative, community-involved data analysis. These principles emphasize the importance of relational and community-involved processes in research with Indigenous communities. While it is impossible to fully capture all the implicit ways in which these principles were integrated, Table 1 illustrates their broad application in the study, such as a focus on relationship building; the formation of a Community Advisory Board (CAB) with Indigenous 2S/LGBTQ+ Elders, advocates and/or people with HIV; attendance at key relationship-building events (e.g., Two-Spirit Powwows, 2S/LGBTQ+ Pride events); community partnerships and accountability; and centering reflexivity throughout the entire investigation.

**Table 1:** Two-Eyed Seeing principles incorporated for enhanced community engagement and culturally attuned research.

Two-Eyed Seeing principle	Incorporation
Author positionality	Positionality statements before each participant interview, with community partners, and/or other collaborators; ensuring reflexivity throughout the entire investigation by memo-taking and employing narrative analysis methods.
Group interpretations and guiding principles	Abiding by the four Rs: respect, relevance, reciprocity, and responsibility when engaging with tribal partners.
Relationship building	In-person attendance at Two-Spirit powwows, prides, and community partner events; continued relationship building by regular check-ins.
Inclusion of indigenous advisory committees and knowledge holders	Formulation of a Community Advisory Board (CAB) to guide culturally responsive research approaches.
Continued community guidance	CAB engagement at key touchpoints in the study; presenting preliminary findings to community members.
Community-involved data analysis	CAB directly engaged in the preliminary analyses of the findings.
Traditional techniques	Land acknowledgements delivered, focus on relationality, and Pendleton blanket gifting to participants who complete the study.

#### 2.2.1 Relationship building

Relationship building was a central priority in the application of the Two-Eyed Seeing approach, as establishing strong, meaningful connections between researchers and Indigenous partners and communities is essential for conducting mutually beneficial and culturally appropriate Indigenous health research (32). Recognizing the importance of being present within the community, the first author prioritized engaging with Indigenous 2S/LGBTQ+ communities through key cultural events, such as the San Francisco Bay Area American Indian Two-Spirits (BAAITS) Powwow and the annual International Two-Spirit Gathering. The first author’s attendance at these events enhanced participant engagement and provided a platform for building rapport with potential study participants. Additionally, the first author’s involvement in the Two-Spirit Dry Lab allowed them to engage with Two-Spirit researchers, leaders, Elders, and activists, creating spaces for meaningful interactions and collaboration. Through these ongoing efforts, the first author fostered early connections with the Two-Spirit community, which fostered trust and collaboration throughout the study.

#### 2.2.2 The formulation of a community advisory board

Establishing a CAB was an initial priority in applying the Two-Eyed Seeing approach to this project. These early relationships developed organically, stemming from the first author’s membership in the Two-Spirit Dry Lab and existing tribal community connections. As part of these early efforts, the first author often shared their “coming in” story as an Indigiqueer Chickasaw researcher with potential CAB members. The term “coming in,” as defined by Wilson (33), refers to a process unique to Indigenous 2S/LGBTQ+ people, describing the act of returning to fully embrace one’s identity and re-establishing a valued place within families, cultures, and communities. This contrasts with the Western concept of “coming out,” which has been shaped by colonial legacies of homophobia, transphobia, and queerphobia in the lands now known as the United States and Canada. Once personal stories were exchanged, the first author extended a verbal and email invitation to join the CAB.

While the first author shared some identities with the CAB members, they also recognized the complexities of insider and outsider roles and sought to occupy insider and outsider positions simultaneously rather than viewing them as oppositional (34). This perspective also aligned with the core principles of the Two-Eyed Seeing approach, which values the integration of multiple perspectives in health research (35).

#### 2.2.3 Community advisory board involvement

The early focus of the study was developing a culturally adapted life review (36), created in close collaboration with the CAB. These initial meetings were crucial in creating a research instrument that allowed participants the autonomy to share their stories in a culturally safe and respectful manner. The CAB encouraged the first author to explicitly situate themselves within the interview process by using positionality statements during interviews and outreach. This practice helped clarify the first author’s dual role as a doctoral student researcher and Indigenous community member. Additionally, the CAB recommended more challenging questions (e.g., “What is the hardest thing about aging?”) to be balanced with more positive, affirming questions (e.g., “What excites you about aging?”). The opening interview question, “Tell me your story of HIV,” was also crafted during a CAB meeting, ensuring the question was grounded in the experiences and values of the participant. The semi-structured interview guide was treated as a living document, continuously refined throughout the study period based on CAB input and participant interview observations. The first author regularly reported to the CAB, allowing for ongoing feedback and adjustments.

Moreover, the CAB played a critical role in participant recruitment by sharing the study through their personal networks to facilitate snowball sampling. Finally, the CAB contributed to community-involved data analysis, engaging in discussions to refine emerging themes and ensuring the analysis reflected the perspectives and values of the community. This collaborative approach was pivotal in ensuring the study remained firmly rooted in Indigenous knowledge, practices, and interpretations sensitive to community needs.

#### 2.2.4 Forging community partnerships

Community partnerships were critical to the successful implementation of the Two-Eyed Seeing approach. In alignment the spirit of Two-Eyed Seeing, both Indigenous-specific and non-Indigenous community organizations were involved in the study, with five community partnerships established through this project by the end of the study period. These partners supported recruitment by posting study flyers at their facilities, sharing about the study through email listservs, and promoting infographics on social media platforms. The first author engaged with community partner contacts through Zoom meetings and, when possible, in-person at conferences, fostering authentic relationships.

## 3 Findings

### 3.1 Hard-to-reach or simply left in the shadows?

The successful recruitment of 15 Indigenous 2S/LGBTQ+ people with HIV from over 17 distinct Indigenous Nations within the first 8 months of the study highlights the central role of community relationships and relationality—principles often overlooked in research with Indigenous communities. Intentional community engagement, support from the CAB, and word-of-mouth recruitment are all attributes that facilitated the steady recruitment of participants. These efforts were enhanced by a commitment to reflexivity, transparency about the study’s goals to benefit the community, and *meeting participants where they were* while employing active listening techniques and empathetic engagement. Two-Eyed Seeing ultimately helped foster trust and cultural resonance, which were key to building meaningful connections with participants.

This successful recruitment process challenges the common narrative that these communities are ‘hard-to-reach populations’ (37). Many participants expressed they were not difficult to engage but instead felt ignored by researchers–particularly non-Indigenous researchers–who failed to make genuine efforts to build relationships with their communities, pointing to the need for more Indigenous researchers. Participants emphasized that this lack of outreach left them feeling invisible, like “*a shadow*.” This sentiment is underscored by a participant who shared:

*I want to say thank you for advocating for Indigenous Two-Spirit. I have done some work nationally, and I realize how much of a shadow we are. You know, there’s no one prioritizing us, especially in the HIV area. Some do Two-Spirit work, but there’s not many advocates in the HIV area, so I want to say thank you for doing this. I’m very honored to take this survey… it’s always an honor to get a referral from [Community Advisory Board] member herself.* (40s, Two-Spirit, transgender participant with HIV for approximately 15 years)

This gratitude underscores the impact of culturally grounded and relationship-driven research approaches embedded in the Two-Eyed Seeing approach, reaffirming that centering community voices and prioritizing relationality are not just ethical imperatives, but essential strategies for meaningful engagement. Our CAB members played a crucial role in this success, reaching out to participants in their network directly and offering valuable guidance on culturally significant events to attend, which facilitated participant engagement.

Authentic community-research partnerships, which are essential for improving research in tribal communities (38), require key actions such as mutual respect, trust, transparency, and active engagement including study updates, ensuring that the community partner is adequately updated on the study, and their needs and perspectives are prioritized throughout the research process.

### 3.2 Meaningful mentorship for the researcher

CAB involvement extended beyond logistical support, providing crucial mentorship to the first author. For an Indigenous doctoral scholar, this mentorship was not just beneficial, but transformative, significantly enhancing the strengths-focused positioning of the research and deepening the first author’s connection to their Indigenous community. For example, the first author used the term “vulnerable” to describe Indigenous 2S and LGBTQ+ PLWH during a meeting in which a CAB member reframed stating:

*As you know we come from a long line of people who have survived 532 years of colonization, every breath I take is an act of survival and every exhale is an act of resistance - or survivance! I and my community are hardly “vulnerable.”* (CAB Member)

This perspective not only reshaped the researcher’s understanding and framing of the research project, but also reinforced the strength and thrivance (39) inherent within Indigenous 2S/LGBTQ+ people with HIV that would not have been actualized without CAB involvement.

### 3.3 Advantages of reflexivity

The integration of reflexivity into the interviews, remaining explicit about the objectives and intentions of the study, proved highly advantageous, particularly in addressing traditional barriers between the researcher and participants and mitigating inherent power dynamics. After the researcher provided their positionality statement and invited questions at the beginning of the interview, one participant shared:

*I was interested in engaging [in this study] because I saw that it was being conducted by a Two-Spirit person, and that it was going to directly benefit IndigiQueer and 2S/LGBT people. So, when I see those kinds of things align, it’s like* “Okay, you have my attention, and this is something that I contribute to.” (40s, Two-Spirit, male participant with approximately 11 years with HIV)

This level of transparency and openness fostered a sense of trust between the researcher and the participants, enabling more meaningful engagement in the research process. Many participants also became advocates for the study, promoting it within their networks. This word-of-mouth endorsement was instrumental in the successful recruitment of additional participants across geographies, underscoring how reflexivity and critical relationality can enhance trust and community engagement within Indigenous research contexts (40).

## 4 Discussion

Qualitative findings from participant feedback, CAB insights, and recruitment success suggest the Two-Eyed Seeing approach effectively fostered culturally responsive practices and engaged a ‘hard-to-reach’ population as perceived/experienced by Western researchers. Applying Two-Eyed Seeing principles enhanced the current research through actions such as generating culturally attuned research questions, the formation of a CAB, co-designing research tools, collecting and interpreting and translating data, participating in community events, and engaging in radical reflexivity to interrogate implicit assumptions and address researcher positionality and biases. These practices promoted relational accountability, and facilitated a deeper connection between the study team, CAB members, participants, and community partners. Applying the Two-Eyed Seeing principles also created safer spaces for community members to share their stories. For example, many participants expressed gratitude for the opportunity to share their stories candidly and to be met without judgement or pathologizing.

Two-Eyed Seeing offers a pathway for Indigenizing contemporary CBPR approaches by more intentionally centering Indigenous research paradigms, methodologies, and worldviews in ways that are responsive to Indigenous people and communities. Such an approach may create opportunities for *repowering* 2S/LGBTQ+ people and communities or recognizing and affirming the inherent power Indigenous peoples already hold to determine what best serves their communities, while drawing upon Indigenous cultural knowledge, practices, and wisdom (41). The potential of *repowering* as a guiding orientation for Indigenous health research warrants further exploration.

Two-Eyed Seeing also offers an important framework for embedding more culturally responsive and equitable practices within clinical and healthcare settings serving Indigenous communities, particularly within the United States. Two-Eyed Seeing offers a health care approach that honors Indigenous health equity and effectiveness for Indigenous peoples by integrating Indigenous knowledge systems, beliefs, values, healing practices, medicines, and understandings of wellness alongside Western biomedical approaches. Within Indigenous health contexts, this may include incorporating practices such as talking circles, storytelling, traditional songs, dance, drumming, smudging, sweat lodges, cold-water ceremonies, and land- and water-based activities including fishing, hunting, harvesting, and the sharing of traditional foods. Models grounded in these approaches have demonstrated promise within clinical settings such as the First Nations Health Authority (FNHA) in Canada (8). Insights from this study suggest that similar efforts to operationalize Two-Eyed Seeing within US healthcare systems may help foster greater cultural safety, relational accountability, trust, and engagement among Indigenous 2S/LGBTQ+ people with HIV. Future research would benefit from more directly examining the tensions within Two-Eyed Seeing frameworks, particularly how efforts to integrate Indigenous and Western approaches in clinical and healthcare settings may inadvertently position these knowledge systems as equally situated without fully acknowledging the colonial histories, structural inequities, and ongoing power imbalances that continue to shape Indigenous health and healthcare experiences.

## 5 Conclusion

Two-Eyed Seeing has emerged as a valuable approach in health sciences research with Indigenous communities, offering a stance through which diverse knowledge systems can converge. Two-Eyed Seeing asserts that both Indigenous and Western knowledge systems offer partial perspectives, and by integrating them, individuals can achieve a richer, though still incomplete, understanding of the world (18). Central to Two-Eyed Seeing is its capacity to prompt critical self-reflection within Western paradigms, urging researchers to re-examine their perspectives and practices to better engage with and respond to the priorities of Indigenous 2S/LGBTQ+ aging with HIV. This ongoing process of self-reflection is crucial for advancing the Indigenization of positivist research (42). Without it, meaningful application of Two-Eyed Seeing remains limited. Future HIV research in Indigenous communities, especially within the US context, should focus on exploring how Two-Eyed Seeing can foster culturally responsive methodologies. By integrating Two-Eyed Seeing, researchers can advance health equity, strengthen community well-being, and improve collaborative partnerships in Indigenous health research, including in the context of HIV.

## Data Availability

The raw data supporting the conclusions of this article will be made available by the authors, without undue reservation.

## Footnotes

1 To “invisibilize” refers to the systemic silencing, exclusion, and marginalization of certain people and communities within society. When social structures—or even the implicit biases of helping professionals—fail to recognize or respond to a group’s distinct lived experiences, histories, cultural contexts, and humanity, that group becomes invisibilized (43).
